# Supplementary material for: Are carbon emissions trading and green financial instruments synergistic? -Comprehensive quantitative research based on content analysis
Source: PLoS One. 2024 Mar 7;19(3):e0298601. doi: 10.1371/journal.pone.0298601 (PMC10919601; doi:10.1371/journal.pone.0298601)
Supplement: S1 Data — (ZIP) [file pone.0298601.s001.zip › Data/地级市Z_F工作报告.zip!/╕ú╜¿/╚2├≈╩╨/└┤╫╘╣1⁄2╓┌║┼í╛┬φ┐╦╩2╛▌═°í┐_2011.docx]

各位代表：

　　现在，我代表市人民政府向大会报告政府工作，请连同《三明市国民经济和社会发展第十二个五年规划纲要（草案）》一并审议，并请市政协各位委员和其他列席人员提出意见。

　　一、2010年和 “十一五”时期经济社会发展回顾

　　2010年，我市各级政府在省委、省政府和市委的正确领导下，认真贯彻党的十七大、十七届三中、四中、五中全会和胡锦涛总书记等中央领导同志来闽考察重要讲话精神，深入贯彻落实科学发展观，全力推动科学发展、跨越发展，经过全市人民共同努力，较好地完成了市十一届人大五次会议确定的目标任务。初步统计，全市地区生产总值972.71亿元，增长13.9%；财政总收入82.16亿元，增长22.3%，其中，地方级财政一般预算收入49.64亿元，增长30.8%；全社会固定资产投资847.81亿元，增长25%；按可比口径实际利用外商直接投资3亿美元，增长15%；外贸出口11.27亿美元，增长47.9%；社会消费品零售总额 229.53亿元，增长18.8%；城镇居民人均可支配收入18194元，增长10.3%；农民人均纯收入6949元，增长9.8%；居民消费价格总水平上涨3.4%；城镇登记失业率2.72%；人口自然增长率6.2‰；单位生产总值能耗降低和二氧化硫、化学需氧量减排任务预计可以完成。一些重点领域和项目取得突破：

　　——永（安）武（平）高速公路建成通车，南（平）三（明）龙（岩）快速铁路三明段、中心城市快速通道、三明沙县机场开工。

　　——新增矿产品深加工、化工和建筑业3个产值超百亿元产业集群，冶金及压延、林产工业产值均突破200亿元。中国重汽福建海西汽车等一批重大项目动工，三明核电公司成立。三明台商投资区、现代物流产业开发区获批为省级开发区。

　　——“通心白莲”、“明健盛”2枚涉农商标首次被认定为中国驰名商标，市级农业龙头企业产值突破100亿元。

　　——中国丹霞·泰宁列入世界自然遗产名录；成功申办2012年第25届世界客属恳亲大会，宁化客家祖地祭祖习俗列入国家非物质文化遗产名录。

　　——三明农村商业银行获银监会批准筹建，全市担保机构贷款担保总额突破100亿元。

　　——三明籍运动员在第16届亚运会、2010年青少年奥运会、世界举重锦标赛上共获6枚金牌。

　　——落实家庭经济困难学生资助政策，惠及学生39.89万人次。大田、建宁、宁化、清流、明溪县全面实施新型农村社会养老保险。市老年活动中心建成并投入使用。

　　过去一年的主要工作和成效是：

　　（一）把握机遇，全力推动跨越发展。突破县域行政区划界限，设立覆盖三明市区、沙县、永安等中心城市发展战略规划区和将乐经济开发区积善园的海西三明生态工贸区，被列入全省10个新增长区域。我们一手抓规划，明确生态工贸区发展目标、功能定位、产业布局；一手抓项目，着力实施总投资920亿元的66个重点项目，完成投资138亿元，已有8个项目投产。

　　按照省里部署，组织大干150天、打好“五大战役”，共实施项目491个，完成投资272.74亿元，重要区域发展、重点建设、重大项目取得突破，城市和城镇面貌得到改观，民生项目顺利完成。开展“项目推进年”活动，组建16个重大项目工作组，163个市级重点项目完成投资179.55亿元，71个项目投产或部分投产。抓紧实施公路交通发展行动计划，加快铁路规划建设，永（安）宁（化）、建（宁）泰（宁）高速公路和向（塘）莆（田）快速铁路三明段加快建设，厦（门）沙（县）、永（安）漳（州）、湄洲湾至重庆高速公路三明段和浦（城）建（宁）龙（岩）梅（州）、长（汀）泉（州）快速铁路三明段前期工作持续推进，普通公路和农村公路建设、改造取得进展。围绕项目用好政策，共争取专项资金、财政转移支付资金66.16亿元，其中专项资金44.86亿元，增长46.6%。争取的资金中用于民生保障26.45亿元，增长65.3%。深化集体林权制度改革，开展明晰产权“回头看”，林权证到户率达75%；推动农村土地承包经营权流转，新增流转面积3.2万亩。三农公司股票恢复上市，省一建公司改制稳步推进。加快行政事业单位资产整合营运，120个市直行政事业单位740处资产全部划拨到市行政事业资产营运中心，原租赁合同到期重新招租或延租后，租金提高50.2%。公共资源交易市场化运作取得实效，市公共资源交易中心的交易额达到37.86亿元，实现增收节支7.33亿元。市级政府机构改革启动，事业单位绩效工资改革全面实施。自主创新力度加大，27个项目列入国家科技计划，新增8家国家高新技术企业，三明国家大型机械装备高新技术产业化基地列入国家高新技术产业化基地，成立6个产业技术创新联盟，“6·18”合同项目数、总投资继续走在全省前列。

　　强化与中央属企业合作，在福建省与央企项目合作洽谈会上签约8个项目，总投资621亿元。成功举办第六届海峡两岸林业博览会，开展“沙县小吃台湾行”，举办海峡两岸乡镇对口交流会，新批办台资企业和合同台资分别增长50%和120%。组织参加“9·8”等招商活动，增强利用外资实效；开展经济协作项目321个，引进内资88.5亿元。外贸进出口势头良好，出口总额突破10亿美元。落实鼓励民间投资政策，民间投资占城镇固定资产投资比重比上年提高8.1个百分点，非公有制企业实现税收增长37.3%，商会经济加快发展。

　　（二）突破重点，发展方式加快转变。以工业增量提质为核心，实施振兴工业行动计划，推动重点产业集聚，八大产业产值1200.51亿元，增长25.1%，占规模以上工业产值的90.3%。出台扶持培育百亿十亿重点工业企业政策措施，新增4家产值超10亿元企业，4家企业列入省培育和壮大的百亿大企业名单，规模以上工业经济效益综合指数249.5%，比上年提高48.9个百分点。加快重点园区开发，新增开发面积1.32万亩，新落地项目133个，新增投产项目95个。以培育战略性新兴产业为方向，加快发展生物医药及生物、新能源、新材料和节能环保产业，生物医药及生物产业实现产值43.6亿元，增长37.8%，天尊不锈钢复合板项目投产，三明核电项目列入国家核电中长期规划，武汉凯迪生物质能源项目动工建设。以壮大现代服务业为重点，落实加快发展流通业行动计划，完成服务业投资396.16亿元，海西金属材料制品市场、三明城市物流园等项目成为新的经济增长点，三明“陆地港”等项目加快建设，红星美凯龙等项目落地三明。持续拓展消费市场，消费对经济增长的贡献率达到28.5%。房地产市场持续平稳发展，投资增长26.8%。实施旅游发展总体规划，一批旅游项目取得突破，新评定四星级旅游饭店3家,游客接待量、旅游总收入持续增长。世界客家始祖文化园、闽台（永安）文化创意产业园等文化产业项目扎实推进。以调整农业结构为基础，落实发展现代农业行动计划，全年粮食总产量111.96万吨，再生稻高产栽培技术全国领先，十大特色农业产业加快发展，农业产业化经营总额达387.05亿元。新增35个无公害农产品、绿色食品、有机食品，“安砂淮山”获准成为国家地理标志保护产品。以节能减排和生态建设为抓手，关闭61家“五小”企业，淘汰330万吨落后水泥产能，25家企业通过清洁生产审核，永安市成为全国林业低碳经济综合试点城市。关闭或搬迁禁养区内219家畜禽规模养殖场，完成52家企业废水深度治理任务；投入1.35亿元，铺设污水管网80.08公里，实现县县有污水处理厂。完成造林绿化面积50.87万亩，生态示范创建走在全省前列。连续11年实现耕地占补平衡。

　　（三）持续拓展，区域经济实力增强。按照“大三明”思路，加快实施中心城市发展行动计划，推进三明市区、沙县同城化及其与永安市的一体化，编制完成三明中心城市空间发展战略规划和市区南部、北部新城控制性详细规划。落实加快市区经济发展政策措施，市区生产总值226.24亿元，占全市的23.3%；地方级财政一般预算收入17.71亿元，增长29.5%。启动贵溪洋新区建设，推进徐碧新城、下洋中区、台江片区开发，三明体育中心、台江大桥、江滨路和西江滨路改造、城市立面改造等项目加快建设或建成，城市建筑风格与色彩逐步显现。

　　提高县域经济发展水平，县城新区开发和旧城改造有序实施，公共设施不断完善，特色与品位提升。特色经济加快发展，形成农产品和资源深加工、生物医药等优势产业,东南翼生产总值和地方级财政一般预算收入分别增长14.4%、32.1%，西北翼生产总值和地方级财政一般预算收入分别增长13.9%、32%。加快小城镇和新农村建设，2个省级综合改革建设试点镇和17个市级中心乡镇规划建设有序推进；22个乡镇、220个村通过省级农村家园清洁行动验收，泰宁县朱口镇被授予全国环境优美乡镇。落实老区苏区比照执行中西部政策，争取中央和省级财政专项资金1.09亿元，比上年增加4600万元。加大扶贫开发力度，投入库区扶持项目资金1.28亿元，库区移民生产生活条件不断改善。

　　（四）组织有力，抗洪救灾与恢复重建成效明显。去年，我市连续遭受冰雹、霜冻和特大暴雨灾害，在省委、省政府坚强领导下，市委、市政府始终把人民群众的生命安全放在首位，周密部署、科学组织、落实责任，全力转移危险区域的群众39.3万人，妥善安置受灾群众2.63万人，把灾害造成的损失降到最低程度。灾后迅速抢修基础设施，组织恢复生产，在较短时间内抢通供水、供电、道路、通讯、广电等设施，恢复工农业生产。同时，在福州市对口支援下，加快农村住房等灾后重建步伐，春节前重建户可搬入新居。

　　（五）突出统筹，公共服务得到加强。创建国家级创业型城市工作取得成效，新增城镇就业2.7万人，农村劳动力转移就业5.14万人，高校毕业生就业率达85%。扩大社会保险覆盖面，实行工伤、生育、医疗保险市级统筹，启动社会保障卡项目。加大住房保障力度，竣工经济适用住房2506套，新开工廉租住房1582套。城乡低保、农村五保补助水平提高。稳定蔬菜等副食品价格，市场供应得到保障。

　　义务教育均衡发展取得新成效，“双高普九”达标县（市、区）达到9个，90%以上进城务工人员子女在公办学校就读。组建市高级技工学校，三明学院艺术设计专业入选国家级特色专业建设点，三明职业技术学院通过教育部高职院校人才培养工作评估。出台进一步加强人才工作意见，一批创业创新人才和团队受到省委、省政府表彰，新增南生科技公司博士后科研工作站。公众科学素质提高。推进医药卫生体制改革，基层群众住院平均医药费用大幅下降，市第一医院“三级甲等”复评名次居9个设区市首位，台江医院动工建设。统计服务质量提高，第六次全国人口普查工作扎实开展。完成农村电影放映工程，市新图书馆、艺术馆、青少年宫和博物馆完成设计、选址，成立三明学院海峡动漫学院。深化出生人口性别比专项治理，开展免费优生检测，低生育水平保持稳定。重视发展老龄事业，永安市被评为全国老龄工作先进单位。民族宗教、水文、气象、防震和地方志工作取得新进展。

　　完善村（居）民自治，基层民主扩大，普法工作加强。群众性精神文明创建活动深入开展，文明城市创建工作取得新成效，未成年人思想道德建设在全国测评中获得佳绩。有效化解矛盾纠纷，清理涉法涉诉信访积案，建立预防和处置医患纠纷“五位一体”长效机制，推行基层综治信访维稳“1+N”联动模式，深入开展严打整治行动，在全省9个设区市党政领导落实综治责任书检查考评中获第一名。强化生产、交通、校园、消防等安全，防范重特大安全事故发生。双拥共建、人民防空和国防后备力量建设得到加强。

　　（六）兑现承诺，惠民实事如期完成。认真落实为民办实事项目和事关群众利益十项实事，教育卫生方面，实施中小学校舍安全重建项目108个，新建36个农村寄宿制学校项目，新建6所公办幼儿园；完成3个社区卫生服务中心和12个乡镇卫生院改扩建。文体和公共设施方面，改扩建20个乡镇综合文化站，新建381家农家书屋；新建323个农民体育健身点；向公众免费开放仙人谷国家森林公园；新增和更新公交车110辆，新建27座标准公厕、4座简易公厕；市区新增4229个停车泊位，贵溪洋公交综合停车场投入使用。社会保障方面，建设16所农村敬老院和2个县级社会福利中心，建成40个社区居家养老服务中心（站）；全面完成助残工程，“光明行动”、特殊教育学校提升、残疾人托养、残疾儿童康复等项目完成年度任务，规划建设市残疾人综合服务中心。强农惠农方面，解决农村24.05万人饮水安全问题；新建5675口农村户用沼气池；森林生态效益补偿标准从原来每亩每年7元提高到12元；完成339个农家店和4个副食品生产基地建设；新建(修)148个村级综合服务场所，农村社区综合维修服务体系建设完成年度任务；开展农村实用技术远程培训15.63万人；改造升级农村公路520公里，实施安保工程2500公里；完成28座水库除险加固；造福工程搬迁10096人；政策性农业保险试点完成年度任务；村级移动服务网点全覆盖。和谐平安方面，食品、药品安全得到保障；建立自然灾害避灾点690个；继续帮助驻明部队建设生产生活项目；我市援建彭州市任务提前完成。

　　（七）注重实效，行政效能不断提高。持续建设开明、服务、法治、廉洁政府，围绕跨越发展强化服务，开展“机关作风建设年”活动,加大治庸治懒力度，帮助357家企业解决房产证、土地证办理问题。改善投资环境，2009—2010年度福建省投资环境满意度测评报告显示，我市投资环境总体满意度居海西20个城市第一。围绕重点工作强化落实，完善责任分解、督查跟进、进度报告制度，将市十一届人大五次会议通过的《政府工作报告》，细化成106项重点工作，全力组织实施，在这次市“两会”上书面报告落实情况。围绕接受监督强化依法行政，出台重大行政决策听取意见和听证制度，加强政府层级监督和行政监察、审计监督；认真执行人大及其常委会决议、决定，办理184件人大代表建议和319件政协提案，自觉接受人大及其常委会的法律、工作监督和政协的民主监督，接受司法监督、社会监督。广泛听取、认真采纳各民主党派、工商联、无党派人士、各人民团体的意见。围绕重要领域强化廉政建设，权力运行进一步规范，工程建设领域突出问题专项治理深入开展，政府投资项目代建制、公示制全面推行，市场中介组织规范发展，廉政建设责任制进一步落实。

　　各位代表，2010年工作任务的完成，标志着“十一五”规划胜利实现。过去五年，是综合实力明显增强的五年，全市生产总值、人均生产总值、财政总收入都比2005年翻番，全社会固定资产投资是2005的5.2倍，城镇化率达到53%；是产业结构加快调整的五年，三次产业比例从22.9∶40∶37.1调整为17∶49.6∶33.4，十大特色农业产业产值占农林牧渔总产值的85%，建成6个产值超百亿元的工业产业集群，高新技术产业产值年均增长94.5%，服务业增加值年均增长14.1%，节能减排目标预计可以完成，生态环境质量走在全国全省前列；是基础设施显著改善的五年，我市被确定为国家公路运输枢纽城市，高速公路通车里程居全省前列，通村公路硬化率达99.5%，2条快速铁路动工建设，在全省率先完成“十一五”新农村电气化建设任务，“六千”水利工程建设取得明显成效；是市区发展空间持续拓展的五年，市区生产总值、规模工业产值、地方级财政一般预算收入均比2005年翻番，市级与县（市、区）联办园区从3个增加到6个，开发面积从1万亩增加到4.05万亩，实现税收从8242万元增加到3.34亿元，市区建成区面积从20.1平方公里增加到27.55平方公里；是民生保障不断加强的五年，城镇居民人均可支配收入、农民人均纯收入年均增长分别为11.7%和10.5%，在全国率先实行新型农村合作医疗补充医疗保险，城镇居民医疗保险全面实施，城乡低保实现应保尽保，实现城乡免费义务教育，公共文化服务体系初步形成，人民群众得到更多实惠。“十一五”发展成就来之不易，这是在省委、省政府和市委正确领导下，在历届政府打下的良好基础上，经过全市上下奋力拼搏取得的。在此，我代表市人民政府，向辛勤工作在各条战线的全市人民，向给予政府大力支持与有效监督的人大代表、政协委员、各民主党派、工商联、各人民团体、无党派人士、离退休老同志、社会各界人士和全体纳税人，向为三明发展做出积极贡献的中央和省属驻明单位、驻明部队、武警官兵、公安民警，向关心支持三明发展的港澳台同胞、海外侨胞、国际友人，致以衷心感谢和崇高敬意！

　　我们清醒地认识到，我市发展还存在不少困难与问题：经济总量不够大，发展方式转变不够快，产业结构不尽合理，第三产业比重偏低，利用外资和出口规模小；资源优势转化提升不快，产业集聚度不高，大项目、好项目不多，科技和人才支撑能力较弱；中心城市辐射带动作用不突出，农村基础设施薄弱，抵御自然灾害能力不强，防灾减灾体系不完善，农民持续增收困难较多；物价上涨压力加大，就业结构性矛盾突出，社会保障体系不够健全，教育、医疗、住房、养老等民生保障与群众需求还有较大差距；人口资源环境压力较大，节能减排和生态环境保护存在薄弱环节，影响安全生产、社会稳定的因素依然存在；一些单位和地方思想不够解放，观念滞后，墨守陈规，办事拖沓。对此，我们将高度重视，采取有力措施加以解决。

　　二、实施“十二五”规划，全力推进三明科学发展、跨越发展

　　按照市委部署，市政府组织编制了《三明市国民经济和社会发展第十二个五年规划纲要（草案）》（以下简称《规划（草案）》），提请大会审议。《规划（草案）》提出了“十二五”期间全市经济和社会发展的指导思想：高举中国特色社会主义伟大旗帜，以邓小平理论和“三个代表”重要思想为指导，深入贯彻落实科学发展观，全面贯彻落实党中央、国务院和省委、省政府加快建设海峡西岸经济区的决策部署，坚持“先行先试、加快转变、民生优先、党建科学”，深入落实“四市”发展战略和“一轴两翼”区域发展思路、三次产业协调推进的产业发展思路，围绕建设海峡两岸交流合作重要平台、海峡西岸经济区重要综合枢纽、海峡西岸经济区先进制造业重要发展区域、海峡西岸经济区重要生态和文化旅游胜地的战略定位，以科学发展为主题，以加快转变、跨越发展为主线，以保障和改善民生为立足点，深化改革开放，加快推进农业现代化、新型工业化和城镇化，深入推进明台交流合作，促进社会和谐稳定，推动又好又快发展，大力提升人民群众的幸福指数，努力把三明建设成为连接沿海、辐射内陆、联动周边的海峡西岸经济区重要增长极，为提前三年全面建成小康社会奠定具有决定性意义的基础。

　　根据上述指导思想，在综合考虑未来发展趋势和条件的基础上，《规划（草案）》确定了我市“十二五”经济社会发展的主要目标：努力实现2015年地区生产总值、财政总收入分别比2010年翻一番，地区生产总值突破2000亿元，财政总收入突破200亿元。经济结构加快优化，人民生活持续改善，社会建设明显加强，生态环境质量保持全省领先，改革开放深入推进。实现上述目标，必须坚持科学发展，按照“好字优先、能快则快，以快促转、转中求进”的要求，推动发展速度、质量、效益相统一，投资、出口、消费相促进，人口、资源、环境相协调。同时，《规划（草案）》提出了今后五年经济社会发展的工作重点和主要任务，我们要着重把握以下五个方面：

　　（一）突出加快转变、跨越发展。加速农业产业化、现代化进程，加快发展特色产业，壮大龙头企业，到2015年建成2个产值超100亿元、3个产值超50亿元、4个产值超20亿元的产业基地。加快工业集群化发展，做强五大主导产业，改造提升传统优势产业，力争到2015年形成1个产值超1000亿元的产业，建成3个产值超100亿元、30个产值超10亿元的大企业。加快发展战略性新兴产业，实现产值年均增长30%以上。大力发展服务业，力争形成2个以上年交易额超100亿元、1-2个超50亿元的专业市场和物流园区，到2015年第三产业增加值占地区生产总值的比重达39%。做大做强建筑业，促进房地产业健康发展。培育壮大旅游业，到2015年游客量和旅游总收入比2010年翻一番。

　　加快建设创新型城市，确保政府对科技创新的引导性投入稳定增长，提高全社会研发经费投入占地区生产总值的比重，完善技术创新体系,加快公共科技服务平台建设，支持企业提高自主创新能力。加强科学普及工作。明显提高财政教育投入，按照优先发展、育人为本、改革创新、促进公平、提高质量、服务大局的要求，推动教育事业科学发展。统筹推进党政人才、企业经营管理人才、高层次专业技术人才等人才队伍建设，到2015年人才总量超过30万人。

　　推进生态市建设，保持森林覆盖率居全省前列，打造生态文明先行区。大力发展循环经济、低碳产业，持续推进节能减排，综合治理城乡特别是中心城市污染，加强城乡饮用水源保护，加快建设资源节约型和环境友好型社会。

　　（二）突出扩大内需、增强后劲。继续强化投资拉动，加大重点产业、基础设施和城市建设投资力度，引导投资向民生和社会事业、农业农村、科技创新、生态环保、资源节约等领域倾斜，力争投资增速超过全省平均水平。实施330个以上列入省、市“十二五”规划盘子的重点项目，提高重点项目投资在固定资产投资中的比重。继续策划、储备、实施重大生产性项目，抓好一批投资10亿元以上的重大工业项目，力争工业投资占固定资产投资比重45%以上。

　　加快建设现代综合交通运输体系，建成中心城市快速通道和永宁、建泰、厦沙、永漳、湄洲湾至重庆三明段等高速公路，力争建宁经宁化至长汀、建瓯经将乐至连城高速公路三明段动工，实现县县通高速公路；建成向莆、南三龙等快速铁路，开工建设浦建龙梅、长泉快速铁路三明段，争取永安至长沙快速铁路项目列入全国铁路网规划；加快三明国家公路运输枢纽建设，尽早实现三明沙县机场通航，力争开通沙县至福州内河航运。动工建设三明核电、永安煤矸石综合利用等项目，推进三明电网跨越提升工程。提升“数字三明”建设水平。建设自然灾害防控体系，提高防灾减灾能力。

　　把增加收入作为扩大消费需求的重要措施，稳步提高政府支出用于改善民生和发展社会事业的比重，进一步提高居民收入在国民收入分配中的比重，提高劳动报酬在初次分配中的比重，提高居民经营性和财产性收入在总收入中的比重，提高低收入者收入，扩大中等收入人群的比重，切实增加农民收入。培育消费热点，落实鼓励消费政策，改善消费环境，促进消费结构优化升级。

　　（三）突出城乡统筹、协调发展。落实主体功能区规划，推进主轴、东南翼、西北翼加快发展，构筑县域特色产业聚集带，力争城镇化率每年提高1.5个百分点。支持苏区、老区、库区和少数民族、边远贫困乡村加快发展，实施老区村跨越发展工程。

　　加快建设三明生态工贸区，打造中部以服务业为主的第三产业核心区、北部以机械装备和现代物流业为主的工贸产业区、西部以新材料产业为主的特色工业集聚区、南部以汽车及机械产业为主的先进制造业先行区，力争地区生产总值年均增长18%以上，到2015年突破1000亿元。

　　发展壮大中心城市，适时推进行政区划调整，向北推动三明市区与沙县同城化，抓紧建设覆盖贵溪洋、洋溪的北部新城，加快开发现代物流产业开发区和金沙园，打造三明市区与沙县的联合中心城区；向南推进与永安市的一体化，加快建设覆盖台江、富兴堡、槐林片区的南部新城，构筑台商投资区、载重汽车城等平台，以产业一体化促进城市一体化；优化市区功能布局，提升城市管理水平。到“十二五”期末，中心城市建成区面积达到100平方公里、人口达到100万。

　　抓好县城规划和建设，合理布局、提升品位，打造一批特色县域城市。抓好省级综合改革建设试点镇和市级中心乡镇建设，带动小城镇发展，加快新农村建设，推进农村环境综合整治，改善农村生产生活条件。加大扶贫开发力度，增强贫困人口自我发展的能力。

　　（四）突出先行先试、激发活力。深化政府机构改革，稳步开展事业单位分类改革，推进财政体制改革，完善国有资产监督管理制度。加快金融服务创新，发展新型金融机构，支持企业上市融资、债券融资。壮大非公有制经济，拓展商会经济，实施明商“回归工程”，做大做强总部经济。坚持和完善农村基本经营制度，深化农村综合改革和集体林权制度综合配套改革，创新农村工作机制。转变外贸增长方式，形成一批大宗商品出口基地和出口龙头企业，提升进口水平。增强招商实效，引导资金、资源、技术、人才投向主导产业等重点领域。加快建设清流国家级台湾农民创业园，办好海峡两岸林业博览会，推进明台产业对接，拓展明台交流交往领域。加强山海协作与区域合作，深化明港澳侨交流合作，支持出国人员回乡创业。

　　（五）突出民生优先、促进和谐。深化创建国家级创业型城市工作，健全创业就业再就业服务体系，完善社会养老、基本医疗、社会救助、保障性住房、社会福利和慈善事业、公共财政投入等社会保障体系。深化医药卫生体制改革，建立覆盖城乡的基本医疗卫生制度，发展中医药事业。加快发展文化事业和产业，健全公共文化服务体系，提高文化产业增加值占地区生产总值的比重。稳定低生育水平，提高出生人口素质，推动人口长期均衡发展。推进广播电视、全民健身和竞技体育事业发展。加强未成年人保护，发展妇女、儿童事业。高度重视民族宗教、老龄工作，创建全国残疾人工作示范城市。

　　加强社会主义核心价值体系建设，深化群众性精神文明创建活动，争创全国文明城市和全国未成年人思想道德建设工作先进城市。以综治工作争创全国“长安杯”为抓手，深化社会矛盾化解、社会管理创新、公正廉洁执法三项重点工作，建立维护群众合法权益机制，完善社会稳定风险评估机制，落实社会治安综合治理责任机制，保持社会安定稳定。自觉接受人大及其常委会的法律、工作监督和政协的民主监督，加强与各民主党派、工商联、无党派人士的联系，支持工会、共青团、妇联等人民团体发挥作用。推进基层民主建设。坚持依法行政，强化廉政建设。支持国防和军队建设，加强民兵、预备役部队建设。

　　三、扎实做好2011年工作，努力实现高位开局

　　今年是“十二五”开局之年。我们要全面贯彻党中央国务院、省委省政府和市委的部署，坚持以科学发展为主题，以加快转变、跨越发展为主线，进一步扩大内需，促进增长，调整结构，统筹协调，深化改革，扩大开放，改善民生，促进和谐，打好“五大战役”，大干开局之年，全力以赴推动三明科学发展、跨越发展。全市经济社会发展的主要预期目标是：全市生产总值增长13.5%；地方级财政一般预算收入增长17%；全社会固定资产投资增长22%；外贸出口增长10%，按可比口径实际利用外商直接投资增长10%；城镇登记失业率控制在3.8%以内；社会消费品零售总额增长18%，居民消费价格总水平涨幅控制在4%左右；人口自然增长率控制在7‰以内；城镇居民人均可支配收入增长12.5%，农民人均纯收入增长12.5%；完成省里下达的单位生产总值能耗降低和主要污染物减排年度任务。围绕实现上述目标，着重抓好以下七个方面工作：

　　（一）打好“五大战役”，确保取得更大突破

　　打好重点项目建设战役。继续开展“项目推进年”活动，强化项目带动，实施186个重点项目，完成年度投资216亿元，开工项目50个以上，实现30个以上项目投产或部分投产。交通、能源方面，永宁高速公路建成通车，加快建设建泰高速公路，动工建设厦沙高速公路尤溪段、永漳高速公路三明段和湄州湾至重庆高速公路的三明至明溪、尤溪中仙至坂面路段，推进建宁经宁化至长汀、建瓯经将乐至连城高速公路三明段前期工作；动工建设三明国家公路运输枢纽，改造提升国省道，续建和新开工省道200公里。加快向莆快速铁路三明段建设，全线开工南三龙快速铁路三明段，动工建设浦建龙梅快速铁路三明段控制性工程，争取长泉快速铁路“可研”获批，推进永安至长沙快速铁路项目前期工作。三明沙县机场完成投资3.5亿元。推进500千伏永安输变电项目前期工作，开工建设一批220千伏、110千伏输变电工程，实施新一轮农村电网改造提升工程。产业方面，开工建设25个以上总投资200亿元的工业重点项目，加快实施57个在建项目，推动15个以上项目建成投产；开工建设6个以上投资超亿元的服务业项目，加快建设12个在建项目。农业、水利设施方面，抓好6个重点水利设施项目，完成11座水库除险加固，确保雨季之前修复损毁的农田、水利设施。抓好10.76万亩农业综合开发土地治理项目，实施土地开发整理3万亩。

　　打好新增长区域发展战役。抓紧制定三明生态工贸区规划，合理布局基础设施、公共设施、产业发展、城市建设项目，实施110个重点项目，总投资1170亿元以上，完成投资160亿元以上，其中，续建项目68个，新开工项目35个，前期项目7个。加快建设9个重点园区，新增开发面积1.8万亩，建成投产51个项目。健全三明生态工贸区开发、建设、管理机制，争取国家、省里政策支持，加大扶持力度，增强对区域发展的支撑力。

　　打好城市建设战役。编制《三明市城市总体规划》，推进各县（市）编制城市近期建设规划。加强市容市貌整治，提高城市精细化管理水平。加快建设中心城市快速通道，完成市区江滨路、西江滨路改造，建设贵溪洋主次干道，实施列东大桥翻建，建成一批公共停车场。加快市区和县（市）城区架空缆线下地。继续改造市区“一河两岸”景观，推进城市立体绿化，建设市区体育公园、南部新城绿地。改造供水管网，加快供气、排水设施建设，完成污水管网铺设。推进旧厂房改造，完善社区基础设施，加强社区管理，建设宜居城市综合体。

　　打好小城镇改革发展战役。继续抓好2个省级试点镇建设，力争新增2个省级试点镇，统筹推进17个市级中心乡镇发展。加快基础设施、公用事业、环境景观建设，实施一批城镇产业项目，落实城乡建设用地增减挂钩、城镇户籍制度改革等政策，促进产业和人口向小城镇集聚。开展农村环境综合整治，进行高速公路、快速铁路沿线以及重要路段、重要节点的村镇改造，推进农村环境连片整治，抓好旧城镇、旧厂房、旧村庄改造，实施造福工程和农村危房改造，今年确定246个示范村，重点抓好水源、家园、田园清洁项目和村容村貌整治、房屋立面改造等项目，改善农村人居环境。

　　打好民生工程战役。就业和社会保障方面，实现全市新型农村社会养老保险全覆盖；新建保障性住房3500套，完成棚户区改造1500套，为在中心城市就业的大学毕业生新建公共租赁房200套；推进养老服务“十百千”工程，实施特殊教育提升工程、“光明行动”、残疾儿童康复等项目，扶持妇女、残疾人、计生家庭创业就业。重大社会事业项目方面，市体育中心投入使用，台江医院完成主体施工，动工建设市图书馆、艺术馆、青少年宫和博物馆、三明革命历史纪念馆、残疾人综合服务中心。医疗卫生方面，实施县乡村三级医疗机构标准化建设，提高新农合和城镇居民基本医疗保险政府补助标准，为城乡居民提供10项免费基本公共卫生服务项目，实施城乡困难居民和儿童特殊病种医疗救助，开展免费孕前优生健康检查和农村孕妇、城市低保孕妇免费产前筛查诊断，提高农村部分计划生育家庭奖励扶助和计划生育家庭特别扶助标准。教育文化方面，逐步实施中职免费教育，今年对中职一年级学生免学杂费，推进农村中小学食宿改善工程，抓好中小学校舍安全工程，实施城乡低保家庭入园幼儿保教费补助，完善普通高中学生国家助学金制度，提高高等学校国家助学金标准，加快幼儿园发展，建设农家书屋，推进农村有线广播“村村响”工程，加快乡镇综合文化站和县（市）城区影院建设，新建300个农民体育健身点，推进市、县两级妇女儿童活动中心建设。强农惠农方面，落实政策性农业保险，建设村级综合服务场所，实施“村村通客车”工程，推动水泥路向农村较大自然村延伸，推进农村家园清洁行动、饮水安全工程，建设农村社区综合维修服务体系，开展农村实用技术远程培训。便民生活方面，抓好“菜篮子”生产基地建设，加快便民商业网点建设，提升公交服务，推进城乡绿化，加快公厕建设，实施城乡通信信息化提升工程。和谐平安方面，治理“餐桌污染”，抓好食品放心工程，建设自然灾害避灾点，推进流域水环境整治与水源保护，深化平安创建，支持驻明部队生产生活和科技拥军、文化拥军项目建设。防灾减灾方面，实施地质灾害防治工程，完善动植物疫病防控体系，抓好气象防灾减灾指挥中心、农村气象灾害预警系统试点等项目，增强重大灾害预警预报、快速反应和紧急救援能力。

　　（二）调整优化产业结构，加快发展方式转变

　　大力发展现代农业。做大做强特色产业，落实粮食生产行政首长负责制，完善粮食生产扶持政策，稳定粮食播种面积，推广粮食优良品种、增产技术，确保粮食生产水平稳定；持续壮大五个农业优势特色产业，扶持30家产值亿元以上龙头企业发展，实现农业产业化经营总额突破400亿元。推广农业“五新”成果，健全农业技术推广体系，加强农业科技队伍建设，加快推进农业机械化，

　　完善新型农业社会化服务体系，推进农民专业合作社规范发展。增加农业农村投入，整合部门涉农资金，财政支出重点向农业农村倾斜，确保总量、增幅、比重均有提高；预算内固定资产投资重点用于农业农村基础设施建设，确保总量和比重进一步提高；土地出让收益重点向农业土地开发和农村基础设施建设倾斜；全面推进村级公益事业一事一议财政奖补试点；通过补助、扶持等办法，引导民间资本投投入农业。拓展农民增收渠道，优化农业种养结构，增加农民生产性收入；及时兑现惠农补贴，增加转移性收入；促进农民就地就近转移就业，鼓励农民进城务工经商，增加工资性收入；引导农民发展多种经营，增加经营性收入；加强对土地承包经营权流转的服务和管理，保障流转收益，增加财产性收入。

　　推进工业集群化发展。推动五大主导产业整合、集聚、延伸，力争产值突破1200亿元；改造提升两个传统优势产业，力争产值达到300亿元。实施52个主导产业和传统优势产业重点项目，特别是加快建设中国重汽海西汽车20万辆商用车项目，力争9月份首台载重汽车下线。全力突破央企对接项目，启动三明核电“三通一平”，力争高效硝基复合肥等项目动工。鼓励企业做大做强，新增2家产值10亿元以上企业。加快园区建设，建成2个产值超100亿元、3个产值超50亿元的园区。

　　培育壮大战略性新兴产业。加快发展生物医药及生物产业，突出抓好一批重点项目，支持龙头企业上规模、上水平，力争产值突破50亿元。着力发展新材料产业，建设小蕉金属新材料产业园，实施不锈钢复合板、轻合金、镍合金、硅系列加工和竹纤维、氟材料、轻纺新型面料等重点项目。积极发展新能源及节能环保产业，推进抽水蓄能电站项目前期工作，建设生物质能源产业项目，抓好垃圾处理装备及资源综合利用等项目，力争一批项目建成投产。

　　加快发展现代服务业。建成海西金属材料制品市场和三明“陆地港”，实施三明城市物流园、国际影视城、建宁闽赣省际物流园等项目，做大金融、会展、中介、服务外包等生产性服务业，加快发展商品贸易、医疗保健、家政服务、社区服务等生活性服务业，新增5家销售额亿元以上商贸物流企业。实施海峡旅游（三明泰宁）产业园等重点旅游项目，增强泰宁世界自然遗产和世界地质公园的品牌带动作用，强化主轴旅游的集散功能，提高安养休闲、生态温泉、客家文化、红色旅游等的吸引力。完善旅游配套设施，办好重点旅游节庆，强化区域旅游协作，力争游客量、旅游收入分别增长25%以上。

　　积极扩大消费需求。把稳定价格总水平放在突出位置，保障重要商品和生活必需品的市场供应、价格稳定、质量安全。建立扩大消费需求的长效机制，改善低收入家庭生活水平。发展新型消费业态，支持餐饮酒店、娱乐健身等行业发展。巩固提升“万村千乡市场工程”，整顿规范市场经济秩序。壮大建筑业，提升建筑业企业。稳定房地产市场，加强市场监管，遏制房价过快上涨。

　　（三）坚持以城带乡，推动区域协调发展

　　加快中心城市建设。加速推进三明市区、沙县同城化及其与永安市的一体化，统筹布局产业、基础设施、公共服务等项目，力争在教育、医疗、通信等方面率先实现同城化，加快共建现代物流产业开发区、台商投资区、载重汽车城、金沙园等园区。抓紧开发北部新城，基本建成徐碧新城，加快建设贵溪洋新区，有序建设洋溪、瑞云等组团；着力抓好南部新城建设，持续推进台江、东霞片区和下洋中区开发；推动沙县、永安重点片区建设。抓好市区城中村、城乡结合部改造，协调中心城市建筑色彩，提高中心城市品位。

　　提升县域发展水平。以县城为重点，集聚工业、商贸、物流等优势产业，加快新区开发和旧城改造，完善配套功能，凸显城区特色，提升县域城市的实力与水平。支持东南翼、西北翼持续发展，依托福银、泉三等高速公路，打造以绿色产业为主的北部产业聚集带，形成以先进制造业为重点的南部产业聚集带。围绕优化规划、完善设施、拓展产业，推进小城镇发展，推动农村基础设施建设，提高新农村建设水平。落实苏区老区发展扶持政策，设立促进苏区老区发展专项基金，实施一批老区村跨越发展工程项目。深入推进扶贫开发，抓好贫困村互助资金试点。支持少数民族乡村发展，做好库区移民安置和后期扶持工作。

　　（四）创新体制机制，提高开放水平

　　继续深化改革。完成林权发证工作，基本实现每户都有林权证；推进集体林权制度综合配套改革，确保继续走在全国全省前列。稳定完善土地承包经营权，鼓励发展适度规模经营，规范土地承包经营权流转市场。继续深化政府机构改革，稳步推进事业单位分类改革。培植壮大财源，强化税收征管，健全财政收入稳定增长机制，深化预算管理制度和国库集中支付、政府采购等改革，完善公共财政体系。稳妥推进省一建公司等企业改制。创新金融服务，尽快实现三明农村商业银行开业运营，引进区外商业银行到我市设立分支机构，探索建立沙县农村金融改革试验区，争取每个县（市、区）设立1家小额贷款公司，力争新增贷款140亿元；组建城市建设投资、交通、旅游、林业等企业集团，支持企业直接融资，壮大担保机构，力争1-2家企业上市。

　　强化自主创新。扶持企业技改提升和科技创新，力争50个以上项目列入国家、省科技计划盘子，新增15家以上企业研发机构、5家以上高新技术企业，新增高新技术产业产值20亿元以上。培育一批拥有核心知识产权的优势企业，力争专利授权量增长20%以上。支持企业争创和经营品牌，参与制定国际、国家和行业标准。力争“6·18”签约项目数、投资额、动工率居全省前列。

　　持续扩大对外开放。用好“9·8”等招商平台，引进一批大项目和配套项目，力争合同项目批办率50%以上。加快重点出口商品基地建设，扶持培育一批出口龙头企业，争创国家级、省级出口品牌，推动加工贸易转型升级。加快建设海峡两岸现代林业合作实验区、清流台湾农民创业园、闽台（泰宁）科技园，抓紧林博园建设，支持三明学院等院校开展对台合作办学，办好第七届海峡两岸林业博览会，扩大明台合作。加快建设沿海产业转移集中区、闽赣边界产业和物流集聚区，提升省级山海协作示范园区。筹备第25届世界客属恳亲大会，加强海外华侨华人及其社团的联络工作，密切与港澳地区、国外友城的沟通联系。

　　壮大非公有制经济。加强政策引导，支持民间资本进入基础产业、基础设施、市政公用事业、社会事业、金融服务等领域。组织参加全省大型民营企业产业项目洽谈会，力争城镇以上民间投资450亿元以上。支持成长型非公有制企业加快发展，力争新增25家产值超亿元企业、60家税收超百万元企业。大力发展商会经济，引进总部经济企业。

　　（五）加强生态建设，促进可持续发展

　　推进生态市建设。实施生态功能区划，加快创建生态县，力争建宁、泰宁、明溪县80%的乡镇达到省级以上生态乡镇标准。加强自然保护区管理，实施生态修复，综合治理水土流失，加大小流域整治力度，建立和完善重点流域、矿产资源、森林资源生态补偿机制。加强水资源开发利用管理，严格河道采砂监管，强化资源开发整合与保护。加快森林资源培育，实施“四绿”工程，确保完成118万亩造林绿化任务。强化耕地保护，实现耕地占补平衡。

　　强化节能减排、环境保护。实施节能“十大工程”，推进30个重点节能改造项目，加快14个循环经济工业示范园区（企业）建设，确保30家企业通过清洁生产审核验收。严把新上项目的产业政策关、资源消耗关、环境保护关，实施40个重点减排项目，加快工业园区污染整治，淘汰“五小”企业，提高污水处理厂营运水平，治理城市噪声和机动车尾气污染。继续强化东牙溪饮用水源保护，动工建设市区第二水源，加快明溪黄沙坑水库等项目建设。

　　（六）强化公共服务，让人民群众共享发展成果

　　做好就业与社会保障工作。落实更加积极的就业政策，完善城乡公共就业服务体系，做好重点人群的就业工作，新增城镇就业2.4万人以上，实现农村劳动力转移就业3万人以上。继续提高企业退休人员养老金水平，推进机关和事业单位养老保险制度改革，将未参保集体企业退休人员纳入基本养老保险，探索解决城镇无收入居民养老保险问题。实行失业保险市级统筹，做好社会保障卡发放工作，建立健全被征地农民生活保障工作机制。扩大新农合普通门诊统筹补偿试点，实施重大疾病住院费用补充补偿，解决县以下集体企业职工等困难群众参加医保问题。增加城乡低保补助，将孤儿基本生活费用纳入财政保障范围，改善优抚对象和革命“五老”人员待遇。发展社会福利、残疾人事业，建立健全红十字会备灾救灾体系，成立市慈善总会，推进养老服务体系建设，保障妇女、儿童合法权益。

　　加强教育和人才工作。优先发展教育，加快“双高普九”进程，推进义务教育学校标准化建设，促进义务教育均衡发展；整合中等职业教育资源，支持三明市农业学校创建国家级中等职业教育改革发展示范学校，推进市区职教园区建设；提升高等院校教学质量和科研水平，支持三明职业技术学院创建省级示范性高等职业院校。实施学前教育发展三年行动计划，推进继续教育、社区教育、老年教育、终身教育发展，支持民族教育、特殊教育发展，促进民办教育健康发展，做好进城务工人员子女就学工作。加强师德教育，提高教师队伍素质。制定、落实我市中长期人才发展规划纲要，健全人才激励机制，加强博士后工作站、院士工作站建设，引进一批高层次、紧缺人才和重点领域急需的专业人才，培养一批创业英才。

　　发展社会事业。完善公共卫生服务体系，落实国家基本药物制度，推进公立医院改革，实施住院医师规范化培训和全科医师能力提升计划，培养高层次医学人才，鼓励医务人员在基层服务。加强重大疾病防控和卫生监督工作，发展中医药和妇幼保健事业。稳定低生育水平，加强流动人口计划生育服务管理，综合治理出生人口性别比偏高问题，开展生育关怀行动。完善公共文化服务体系，加强文物和非物质文化遗产保护，争创国家级客家文化生态保护实验区。加快实施文化产业项目推进计划，重点推进世界客家始祖文化园、闽台（永安）文化创意产业园等园区建设，实施海峡闽中文化创意中心等项目，积极拓展数字电视增值业务，打造特色文化产业。发展哲学社会科学，繁荣新闻出版、文学艺术。推进“科普惠农兴村计划”，提高全民科学素质。开展全民健身运动和老年体育活动，加强竞技体育后备人才培养，承办好省第七届农民运动会。继续加强统计工作，夯实统计基层基础，促进民族宗教、档案、史志、水文、防震等工作全面进步。

　　创新社会管理。以“满意在三明”为主题，以“三明三创三促”为抓手，持续深化群众性精神文明创建活动，全力争创全国文明城市。深入推行基层综治信访维稳“1+N”联动工作模式，加强流动人口服务管理和特殊人群帮教管理，推动社区矫正工作，动工建设市看守所。落实信访工作责任制，健全重大项目实施和重大政策制定的社会稳定风险评估机制，落实依法处理信访事项“路线图”，妥善化解和处理矛盾纠纷。强化安全生产“一岗双责”和企业主体责任，抓好重点行业和领域的安全监管，健全突发公共事件应急处置机制。完善村（居）民自治，扩大基层民主。抓好普法教育和法律援助工作。加强与各民主党派、工商联、无党派人士的联系与协商，充分发挥工会、共青团、妇联等人民团体的作用。争创新一轮全国双拥模范城，推进国防教育、国防动员及国防后备力量建设。

　　（七）提高行政效能，增强服务发展能力

　　强化解放思想，建设开明政府。在“学”中解放思想，不断提高学习力，加强积累、注重提升，力求思想紧跟形势发展，观念适应时代要求，工作把握政策导向。在“谋”中解放思想，“十二五”时期我市将全面进入高速公路、快速铁路时代，必须打破思维定势，跳出旧的路径依赖，视野要宽、起点要高、手笔要大，敢谋大事，善谋大项目，促进大开发、大发展。在“干”中解放思想，不能坐而论道、评头论足，要通过具体的平台和抓手，打破常规、高效运作，做到胆子更大、办法更多、步子更快。

　　强化改进作风，建设服务政府。学习李彬等模范人物先进事迹，激发比学赶超的创业热情。深化机关效能建设，强化政府绩效管理和绩效评估，加强效能督查、投诉办理和行政问责。推进审批制度改革，加强行政服务中心建设，加快发展电子政务，推行网上联动审批，完善视频监控系统和审批监察系统。精心组织实施“十二五”规划和“五大战役”，创新运作机制，强力推进，务求必胜。顺应群众改善物质条件、提高健康水平、追求精神生活、维护基本权益等新期待，办好便民利民的实事，解决事关群众安危的问题，让群众生活得更方便、更安稳、更有质量。

　　强化依法行政，建设法治政府。主动接受人大及其常委会的法律、工作监督和政协的民主监督，自觉接受社会各界和人民群众的监督，加强政府层级监督和专门监督，办好人大代表建议和政协提案。深化政务公开和政府信息公开，完善公众参与、专家论证和政府决定相结合的行政决策机制，健全决策风险评估和纠错机制。贯彻国务院加强法治政府建设和推进市县政府依法行政的部署，规范行政执法行为和行政自由裁量权，做好行政复议和规范性文件备案审查工作。

　　强化制度落实，建设廉洁政府。认真执行《党员领导干部廉洁从政若干准则》，严格遵守“52个不准”规定，筑牢思想防线，做到干净干事。落实党风廉政建设责任制，抓好规范权力运行、健全市场机制、完善中介组织等制度建设，健全公共资源交易管理和运行体制，加强惩治和预防腐败体系建设。严格按财经法规办事，压缩行政开支，严肃查办违纪违法案件，树立廉洁为民形象。

　　各位代表，三明发展的前景广阔而美好，人民群众的期待殷切而厚重。让我们紧密团结在以胡锦涛同志为总书记的党中央周围，高举中国特色社会主义伟大旗帜，以邓小平理论和“三个代表”重要思想为指导，深入贯彻落实科学发展观，在市委的正确领导下，团结和依靠全市人民，坚定信心，开拓进取，奋勇前进，推动科学发展、跨越发展，以优异的成绩迎接中国共产党成立90周年！
